# Identification of Potential SARS-CoV-2 CD8^+^ T Cell Escape Mutants

**DOI:** 10.3390/vaccines10040542

**Published:** 2022-03-31

**Authors:** Syed Faraz Ahmed, Muhammad Saqib Sohail, Ahmed Abdul Quadeer, Matthew R. McKay

**Affiliations:** 1 Department of Electrical and Electronic Engineering, University of Melbourne, Parkville, VIC 3010, Australia; faraz.ahmed@unimelb.edu.au; 2 Department of Electronic and Computer Engineering, The Hong Kong University of Science and Technology, Hong Kong SAR, China; mssohail@connect.ust.hk; 3 Department of Microbiology and Immunology, The Peter Doherty Institute for Infection and Immunity, University of Melbourne, Melbourne, VIC 3000, Australia; 4 Department of Chemical and Biological Engineering, The Hong Kong University of Science and Technology, Hong Kong SAR, China

**Keywords:** SARS-CoV-2, COVID-19, CD8^+^ T cell epitopes, immunoprevalent, immune escape, mutations, variants of concern

## Abstract

Memory SARS-CoV-2-specific CD8^+^ T cell responses induced upon infection or COVID-19 vaccination have been important for protecting against severe COVID-19 disease while being largely robust against variants of concern (VOCs) observed so far. However, T cell immunity may be weakened by genetic mutations in future SARS-CoV-2 variants that lead to widespread T cell escape. The capacity for SARS-CoV-2 mutations to escape memory T cell responses requires comprehensive experimental investigation, though this is prohibited by the large number of SARS-CoV-2 mutations that have been observed. To guide targeted experimental studies, here we provide a screened list of potential SARS-CoV-2 T cell escape mutants. These mutants are identified as candidates for T cell escape as they lie within CD8^+^ T cell epitopes that are commonly targeted in individuals and are predicted to abrogate HLA–peptide binding.

## 1. Introduction

SARS-CoV-2-specific neutralizing antibodies and T cells are generated in response to COVID-19 infection and vaccines. The level of immunity that they provide is challenged by the evolution of SARS-CoV-2 and the rise of new genetic variants such as Omicron (see [1] for details on SARS-CoV-2 variants). This presents a key issue for neutralizing antibodies for which genetic mutations enabling escape have been widely reported (e.g., [2,3,4]). For SARS-CoV-2-specific T cell responses, multiple studies have suggested that they are largely robust to mutations observed in the variants of concern (VOCs) so far [5,6,7,8]. However, in a small fraction of recovered and vaccinated individuals, loss of CD8^+^ T cell responses has been reported against variants such as Omicron [8,9]. Moreover, for some specific CD8^+^ T cell epitopes, it has been reported that mutations have weakened or abrogated T cell recognition in some individuals [10,11]. These recent immunological studies [8,9,10,11] indicate at least some capacity for SARS-CoV-2 to escape T cell responses.

CD8^+^ T cell responses present an important weapon against COVID-19, having been associated with limiting disease severity in infected [12] as well as vaccinated individuals [13]. Mutations that facilitate T cell escape could potentially compromise the protection provided by COVID-19 vaccines and infection-induced immunity against severe disease [14,15]. However, the degree to which circulating SARS-CoV-2 viruses may evade CD8^+^ T cell responses is not yet well understood and requires detailed experimental investigation. Here, we report results to aid such studies by identifying and screening mutations observed among sequenced SARS-CoV-2 viruses that may facilitate CD8^+^ T cell escape.

## 2. Materials and Methods

### 2.1. Acquisition of SARS-CoV-2 Epitope Data

We obtained SARS-CoV-2 CD8^+^ T cell epitope data from the dashboard reported in [5] (https://www.mckayspcb.com/SARS2TcellEpitopes/; accessed on 1 February 2022). This dashboard provides up-to-date information on SARS-CoV-2 epitopes targeted by T cells in COVID-19 recovered individuals. The downloaded data comprised a total of 753 distinct HLA-specific CD8^+^ T cell epitopes.

### 2.2. Acquisition of SARS-CoV-2 Sequence Data

SARS-CoV-2 sequence data were obtained from the GISAID database (https://www.gisaid.org/) accessed on 6 September 2021. Only the complete (full genome) sequences derived from human hosts with high coverage were downloaded. All (2,436,276) downloaded sequences were aligned to the SARS-CoV-2 reference genome (GenBank: NC_045512.2) using MAFFT [16]. The aligned genomic sequences were then translated using an in-house code to obtain the protein sequences. The positions of the open reading frames provided with the reference sequence were used to identify the respective protein regions of the full genome.

### 2.3. Identification of Epitope Mutants

For each SARS-CoV-2 epitope, we screened the processed sequence data at the epitope location and defined an *epitope mutant* as any epitope sequence which differed from the reported sequence of that epitope. Epitope mutants comprising any ambiguous amino acid were discarded. The count of each identified epitope mutant, i.e., the number of sequences comprising the epitope mutant, was also recorded.

### 2.4. Prediction of Peptide–HLA Binding Prediction

To understand if an identified epitope mutant impacts the binding of the associated epitope with its cognate HLA allele, we used the well-known peptide–HLA binding prediction method, NetMHCpan4.1 [17]. All predictions were made using the default parameters of the prediction method.

## 3. Results and Discussion

To identify potential CD8^+^ T cell escape mutants, we analyzed mutations lying within the 753 experimentally determined SARS-CoV-2 epitopes based on ~2.5 million SARS-CoV-2 sequences (Materials and Methods). This analysis revealed over 35,000 epitope mutants across proteins. Comprehensive experimental testing of these mutants for their potential to evade CD8^+^ T cell responses is prohibitive, necessitating further screening. A more refined set was obtained by considering those mutants that are associated with epitopes that are commonly targeted globally and which thereby have the potential to result in widespread T cell escape. A list of 20 such immunoprevalent SARS-CoV-2 CD8^+^ T cell epitopes was reported in [5], which are collectively estimated to be targeted by at least 85% of the global population based on their HLA alleles association [18]. Individually, the global coverage of the HLA alleles associated with the immunoprevalent epitopes ranged from 13% to 39%. An important feature of these epitopes is that T cell responses against them were recorded in multiple studies across different geographical locations and in more than half of the COVID-19 recovered individuals tested [5]. These 20 immunoprevalent T cell epitopes have been remarkably conserved so far and do not comprise any mutation specific to VOCs observed to date [7]. Restricted to this immunoprevalent epitope set, the number of HLA-specific epitope mutants was reduced to 914.

To determine the likely escape mutations within immunoprevalent epitopes, a further screening step can be performed using tools to predict the impact of mutations on peptide–HLA binding (a prerequisite for T cell recognition [19]). For this purpose, we employed NetMHCpan4.1 [17], a computational method whose predictions have lined up well with SARS-CoV-2 experimental studies describing CD8^+^ T cell epitopes [20], as well as those reporting HLA binding of specific T cell epitopes [10,11].

Examination of each mutant of the immunoprevalent epitopes revealed that the majority (744/914) were predicted to have no effect on HLA binding, while very few increased binding (4/914). However, almost one-fifth (166/914) of the epitope mutants were predicted to decrease binding, and hence, these presented candidates for enabling escape from T cell responses against commonly targeted SARS-CoV-2 CD8^+^ T cell epitopes. We recommend that these 166 epitope mutants (listed in Supplementary Material Table S1) be considered for experimental investigation. This may involve conducting assays such as targeted HLA binding or HLA ligand elution assays, in combination with standard T cell assays such as multimer qualitative binding, enzyme-linked immunospot (ELISPOT), or intracellular cytokine staining (ICS) interferon-γ (IFN-γ) release, and activation-induced markers (AIM) assays.

Among the identified 166 epitope mutants, most have been observed so far at only low frequencies within the global SARS-CoV-2 sequence data. Only 83 epitope mutants have been observed five times or more (listed in Table 1). Among these, about half (42/83) are associated with HLA-A*02:01, the most prevalent HLA allele globally. Of the 83 epitope mutants, 16 are derived from the spike (S) protein, which could be particularly important given that most COVID-19 vaccines target the S antigen. These also include a mutant of a notable immunoprevalent epitope, YLQPRTFLL, which has been shown to be targeted during COVID-19 infection in more than 10 different cohorts so far [5]. The remaining 67/83 epitope mutants are derived from SARS-CoV-2 proteins besides S, which are of interest for T cell responses induced by inactivated COVID-19 vaccines or prior infection.

The approach that we have adopted, which screens potential T cell epitope mutants based on their ability to evade peptide–HLA binding, is a conservative strategy. This is because a loss of peptide–HLA binding is irreparable since such a peptide would not be expressed on the cell surface for recognition by T cells. It is also possible that epitope mutants that do not decrease HLA binding may still result in T cell escape, mediated by other mechanisms such as abrogation of T cell receptor (TCR) binding with the peptide–HLA complex [21]. However, T cell escape via this means may be recovered by TCR recognition by other T cell clonotypes present in an individual [22]. Thus, while T cell escape through abrogation of TCR binding should not be ignored, the epitope mutants that we have identified to decrease peptide–HLA binding appear to be clear candidates for conferring T cell escape.

## 4. Conclusions

SARS-CoV-2 may evolve mutations to broadly escape T cell responses. While memory T cell responses induced by prior infection or vaccination have so far mainly remained robust [5,6,7,8], some evidence of T cell escape has been reported [8,9,10,11]. Continued monitoring of the capacity of SARS-CoV-2 to broadly evade T cell responses is required, as widespread T cell escape could compromise population-level immunity and have significant clinical and public health implications. To aid such monitoring efforts, our study considered SARS-CoV-2-specific CD8^+^ T cell responses and sought to identify potential SARS-CoV-2 CD8^+^ T cell escape mutants.

A comprehensive experimental evaluation of all potential SARS-CoV-2 CD8^+^ T cell escape mutants would involve testing all relevant mutations observed in available genetic sequence data, which is prohibitive as it would involve evaluating tens of thousands of mutations within known SARS-CoV-2 epitopes. To help address this challenge, we performed a computational analysis to facilitate targeted experimental screening of T cell escape mutants. The main outcome of our study, given in Table 1, is a selective list of 83 observed SARS-CoV-2 mutations that lie within commonly targeted CD8^+^ T cell epitopes and have been observed in the SARS-CoV-2 global sequence data. An important feature of these epitope mutants is that they are expected to decrease peptide–HLA binding, which is a prerequisite for T cell recognition. Escape from these epitopes could be problematic, as it could affect vaccine and infection-induced T cell immunity in a large fraction of the population.

Overall, our computational study leverages available SARS-CoV-2 sequence and immunological data to direct attention towards commonly targeted T cell epitope mutants that emerge as clear candidates to confer escape from the vaccine or infection-induced responses. These epitope mutants are recommended for immediate experimental investigation.

## Figures and Tables

**Table 1:** List of SARS-CoV-2 immunoprevalent HLA-specific CD8^+^ T cell epitope mutants recommended for experimental investigation.

Epitope ^1^	HLA	Epitope Mutant ^2^	Count
S			
_691_SIIAYTMSL_699_	A*02:01	SII**V**YTMSL	720
		SIIAYTM**L**L	655
		**P**IIAYTMSL	205
		SIIAYTM**A**L	181
		S**F**IAYTMSL	38
		SI**V**AYTMSL	24
		SIIAY**A**MSL	7
		SIIAYTMS**F**	7
		**T**IIAYTMSL	5
		SII**F**YTMSL	5
_1208_QYIKWPWYI_1216_	A*24:02	QYIKWPWY**T**	314
		Q**H**IKWPWYI	15
		QYIKWPWY**S**	13
_1000_RLQSLQTYV_1008_	A*02:01	R**F**QSLQTYV	20
		RLQSLQTY**A**	10
_269_YLQPRTFLL_277_	A*02:01	**C**LQPRTFLL	6
M			
_26_FLFLTWICL_34_	A*02:01	FLFLTWIC**F**	1478
		FLFL**I**WICL	1394
		FLFLT**C**ICL	154
		**L**LFLTWICL	85
		F**I**FLTWICL	33
		**C**LFLTWICL	17
		F**V**FLTWICL	14
		FLFLTWIC**I**	13
		**V**LFLTWICL	8
		FL**L**L**I**WICL	7
N			
_338_KLDDKDPNF_346_	A*02:01	KLDDKD**S**NF	633
		KLD**N**KDPNF	226
		KL**N**DKDPNF	177
		K**F**DDKDPNF	118
		KLD**Y**KDPNF	78
		KLD**H**KDPNF	62
		KL**G**DKDPNF	38
		KLDDKD**Q**NF	35
		KLD**V**KDPNF	13
		KL**E**DKDPNF	11
		KLD**G**KDPNF	10
		KL**A**DKDPNF	6
		KL**V**DKDPNF	6
		KLD**A**KDPNF	6
		KLDDKDPN**S**	5
_361_KTFPPTEPK_369_	A*03:01	K**K**FPPTEPK	300
		KTFPPTEP**N**	107
		KTFPPTEP**I**	57
		K**R**FPPTEPK	30
		KTFPPTEP**E**	16
		KTFPPTEP**T**	14
		KTFPPTEP**L**	6
_361_KTFPPTEPK_369_	A*11:01	K**K**FPPTEPK	300
		KTFPPTEP**N**	107
		KTFPPTEP**I**	57
		K**R**FPPTEPK	30
		KTFPPTEP**E**	16
		KTFPPTEP**T**	14
		KTFPPTEP**L**	6
_134_ATEGALNTPK_143_	A*11:01	A**I**EGALNTPK	9685
		**V**TEGALNTPK	1162
		A**A**EGALNTPK	196
		A**N**EGALNTPK	101
		A**P**EGALNTPK	36
		**T**TEGALNTPK	27
_361_KTFPPTEPKK_370_	A*03:01	K**K**FPPTEPKK	300
		K**R**FPPTEPKK	30
		KTFP**S**TEPK**N**	28
_105_SPRWYFYYL_113_	B*07:02	S**S**RWYFYYL	23
ORF3a			
_112_VYFLQSINF_120_	A*24:02	V**H**FLQSINF	339
		VYFLQSIN**C**	112
		VYFLQSIN**S**	50
_139_LLYDANYFL_147_	A*02:01	L**F**YDANYFL	2582
		LLYDANYF**F**	1276
_207_FTSDYYQLY_215_	A*01:01	FTSDYYQL**C**	121
		FTSDYYQL**H**	64
ORF1a			
_1637_TTDPSFLGRYM_1647_	A*01:01	TT**N**PSFLGRYM	2106
		T**I**DPSFLGRYM	1368
		T**N**DPSFLGRYM	102
		**I**TD**L**SFLGRYM	55
		T**I**D**L**SFLGRYM	5
_1321_PTDNYITTY_1329_	A*01:01	P**P**DNYITTY	141
		P**K**DNYITTY	13
		PTDNYITT**H**	10
_2332_ILFTRFFYV_2340_	A*02:01	I**F**FTRFFYV	696
		IL**C**TRFFYV	10
_1636_HTTDPSFLGRY_1646_	A*01:01	HTTDPSFLGR**H**	5

^1^ Reported as immunoprevalent in [5]. The numbers in the subscript before and after the epitope sequence are the start and end indices of the epitope within the corresponding protein. ^2^ An epitope mutant represents an experimentally determined CD8^+^ T cell epitope with at least one mutation (shown in bold) observed in its sequence. The listed epitope mutants are predicted (using the default parameters of NetMHCpan4.1) to decrease HLA binding and occur at least five times in SARS-CoV-2 global sequence data (~2.5 million). The epitope mutants belonging to a specific protein are grouped together. Within each group, the epitope mutants are presented in descending order according to the number of times they are observed in the global sequence data.

## Data Availability

The authors confirm that the data supporting the findings of this study are available within the article or its Supplementary Materials.

## Supplementary Materials

The following supporting information can be downloaded at: https://www.mdpi.com/article/10.3390/vaccines10040542/s1, Table S1 (included as a separate file): List of SARS-CoV-2 immunoprevalent CD8^+^ T cell epitope mutants predicted to decrease HLA binding.
